# Cytometry meets next-generation sequencing – RNA-Seq of sorted subpopulations reveals regional replication and iron-triggered prophage induction in *Corynebacterium glutamicum*

**DOI:** 10.1038/s41598-018-32997-9

**Published:** 2018-10-05

**Authors:** Raphael Freiherr von Boeselager, Eugen Pfeifer, Julia Frunzke

**Affiliations:** Institute of Bio- und Geosciences, IBG-1: Biotechnology, Forschungszentrum Jülich GmbH, 52425 Jülich, Germany

## Abstract

Phenotypic diversification is key to microbial adaptation. Currently, advanced technological approaches offer insights into cell-to-cell variation of bacterial populations at a spatiotemporal resolution. However, the underlying molecular causes or consequences often remain obscure. In this study, we developed a workflow combining fluorescence-activated cell sorting and RNA-sequencing, thereby allowing transcriptomic analysis of 10^6^ bacterial cells. As a proof of concept, the workflow was applied to study prophage induction in a subpopulation of *Corynebacterium glutamicum*. Remarkably, both the phage genes and flanking genomic regions of the CGP3 prophage revealed significantly increased coverage upon prophage induction – a phenomenon that to date has been obscured by bulk approaches. Genome sequencing of prophage-induced populations suggested regional replication at the CGP3 locus in *C. glutamicum*. Finally, the workflow was applied to unravel iron-triggered prophage induction in early exponential cultures. Here, an up-shift in iron levels resulted in a heterogeneous response of an SOS (P_*divS*_) reporter. RNA-sequencing of the induced subpopulation confirmed induction of the SOS response triggering also activation of the CGP3 prophage. The fraction of CGP3-induced cells was enhanced in a mutant lacking the iron regulator DtxR suffering from enhanced iron uptake. Altogether, these findings demonstrate the potential of the established workflow to gain insights into the phenotypic dynamics of bacterial populations.

## Introduction

Microbial communities are highly complex and undergo dynamic changes in response to environmental conditions. Even clonal populations of bacterial species may display considerable variation at the phenotype level^1^. In recent years, a large set of methodological approaches have emerged, enabling functional single-cell analysis via flow cytometry or live-cell imaging on the basis of fluorescent reporter constructs, antibody labeling and a variety of commercially available fluorescent dyes^2^. Recent approaches now provide insights in microbial communities going beyond established model systems, e.g., by monitoring the assimilation of isotope-labelled nutrients^3,4^. Many of these approaches enable the visualization of microbial phenotypic dynamics at single-cell resolution but obscure the underlying molecular reasons and/or consequences.

By combining cell sorting and state-of-the art omics technologies, global insights into the proteome and/or transcriptome of sorted subpopulations can be gained. The concept of cytomics was, for example, applied to study subpopulations of *Cupriavidus necator* JMP 134 exposed to toxic phenol concentrations^5^. In that study, the time-consuming sorting of 10^9^ bacterial cells was still a significant drawback. More recent studies reported on robust proteomics workflows for the analysis of as few as 1 × 10^6^ cells^6,7^. Whereas significant progress has been made in the transcriptomic analysis of single eukaryotic cells^8–10^, such analysis is still hampered in the case of bacteria due to the low half-life of bacterial mRNAs^11^. Nevertheless, approaches such as dual-RNA revolutionized our understanding of host-pathogen interaction^12,13^. Furthermore, early studies reported on the transcriptome analysis of individual bacterial cells such as *Burkholderia thailandensis* and the cyanobacterium *Synechocystis* sp. PCC 6803^14–16^.

In this study, we have established a workflow for the RNA-Seq analysis of bacterial subpopulations and applied this method for the analysis of sorted cells of the biotechnological platform organism *Corynebacterium glutamicum*. The genome of *C. glutamicum* ATCC 13032 contains three cryptic prophage elements, of which the large prophage CGP3 (187 kbp) undergoes spontaneous activation^17,18^. Furthermore, CGP3 can be induced in an SOS-dependent pathway^19^, e.g., by the addition of the DNA-damaging antibiotic mitomycin C, as well as by a recently reported counter-silencing mechanism based on the overexpression of an N-terminally truncated variant of the prophage silencer CgpS^20^. By the means of fluorescent reporter constructs, cells were isolated via fluorescence-activated cell sorting (FACS). The example of CGP3 induction was chosen as a stable and easily traceable transcriptomic response upon prophage induction. Interestingly, the RNA-Seq analysis of cells undergoing prophage induction provided the first evidence for regional replication at the CGP3 locus. Furthermore, subpopulation transcriptomics revealed an iron-triggered CGP3 induction in the early exponential phase of *C. glutamicum* induced by the cellular SOS response.

## Results and Discussion

### Prophage induction – A test case for the establishment of an RNA-Seq workflow

**Figure 1:**
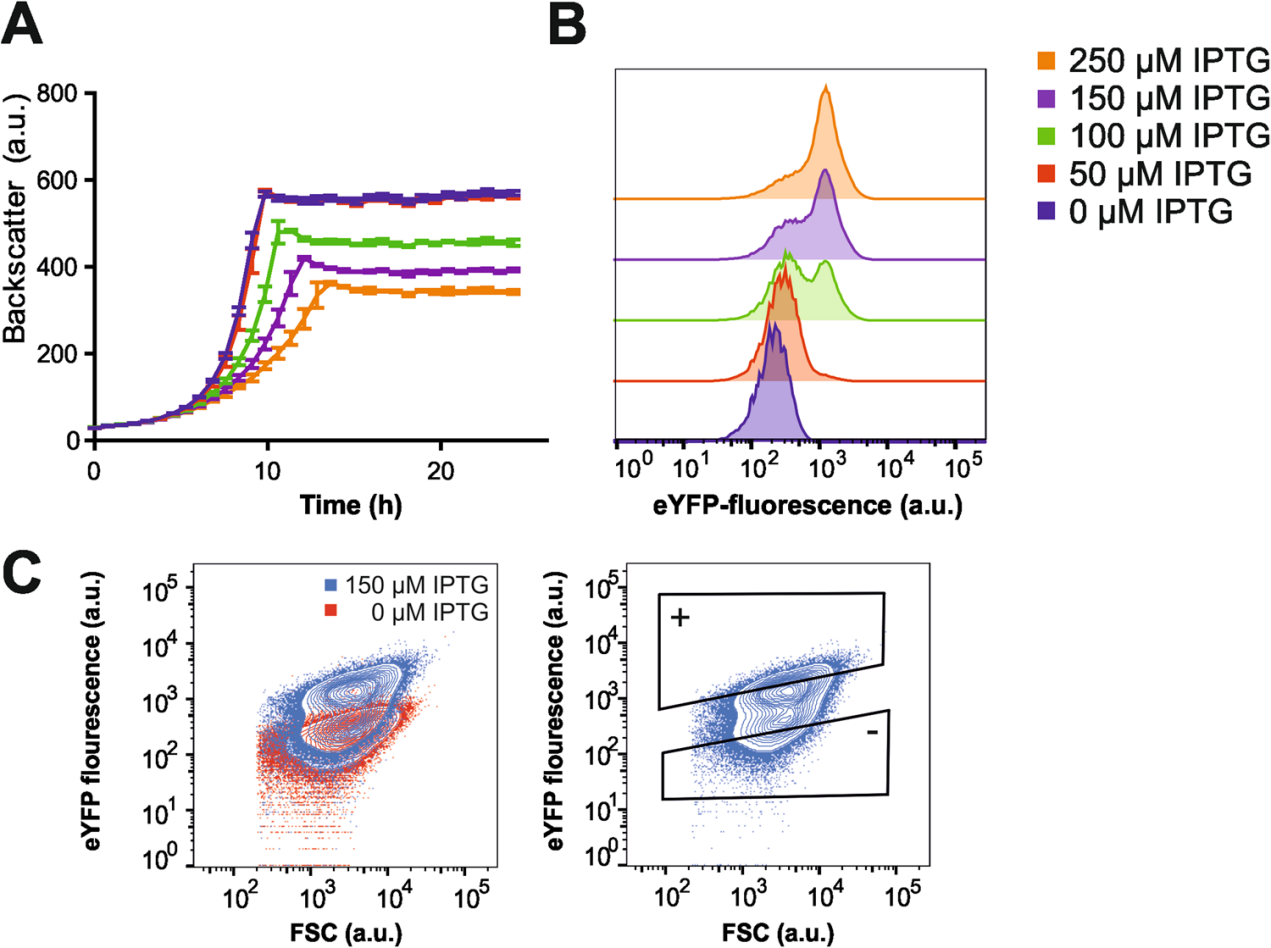
Heterogeneous prophage induction in *C. glutamicum* populations. For proof-of-concept studies, prophage induction was triggered by counter silencing as described previously^20^. Phage induction was visualized by the means of fluorescent protein production using a fusion of the P_*lys*_ phage promoter to *eyfp*. (**A**) Growth of the strain *C. glutamicum*::*Plys-eyfp*/pAN6_*N-cgpS* treated with different IPTG concentrations. (**B**) Flow cytometry analysis after six hours revealed a significant fraction of prophage induced cells upon addition of >100 µM IPTG. (**C**) Contour plots of an induced (blue) and an uninduced sample (red). Gating strategy applied for the isolation of 10^6^ cells from phage positive and negative populations are shown in the right plot.

**Figure 2:**
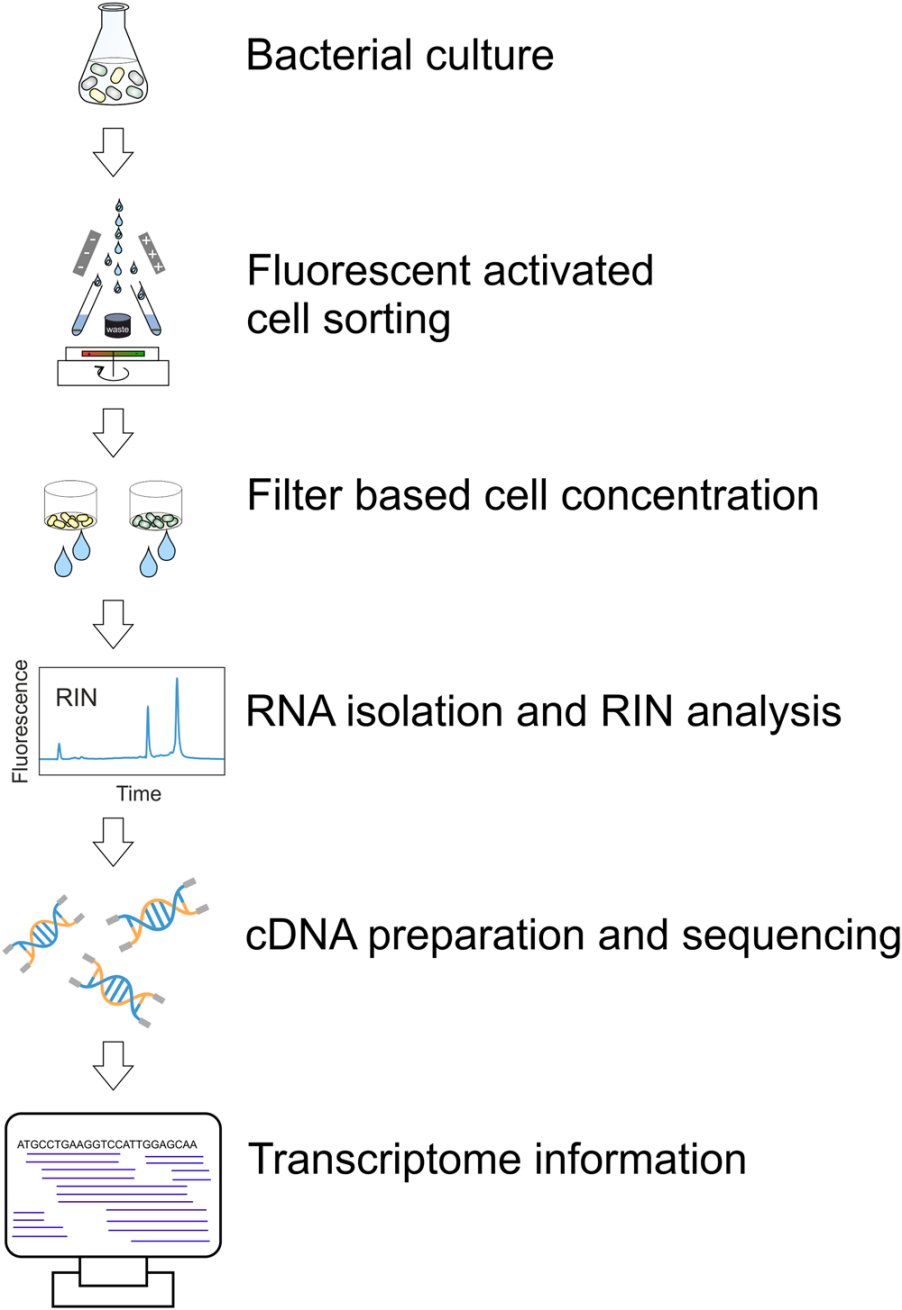
Experimental workflow for the transcriptome analysis of bacterial populations after cell sorting. Cultures were analyzed by flow cytometry and sorted by the means of the fluorescent reporter signal. One million cells were sorted and immediately treated with an RNA stabilization agent (RNAlater or RNAprotect). Subsequently, cells were concentrated on a filter plate, flash frozen in liquid nitrogen and stored at −80 °C. Prior to RNA extraction, the cells were treated with lysozyme and mutanolysine. The quality of RNA was determined as RIN value (>7 for samples used for sequencing). Extracted RNA of appropriate quality was then used for cDNA library preparation and sequencing.

In this study, we were aiming at the establishment of an integrative workflow combining the power of single-cell analysis via fluorescence-activated cell sorting (FACS) with RNA sequencing to unravel the differences between bacterial subpopulations at the level of gene expression. For this purpose, we chose the induction of the large (187 kbp) prophage CGP3 of *C. glutamicum* as a test case providing a distinct readout for workflow optimization. To visualize activation of CGP3 in single bacterial cells, we used a previously designed reporter circuit where the promoter of a putative lysine gene (cg1974) was fused to *eyfp*. By this means, production of eYFP is indicative for prophage induction in the particular cell^19^. By applying the recently described counter-silencing mechanism^20^ (scheme depicted in Fig. S1), we were able to adjust the induction of CGP3 by modulating the expression of the N-terminal oligomerization domain of the prophage silencer CgpS, which was under control of the IPTG-inducible promoter P_*tac*_ (pAN6-N-cgpS). Increasing concentrations of IPTG resulted in an obvious growth defect of the population coinciding with an increased fraction of CGP3-induced cells (Fig. 1). Upon induction with 100–150 μM IPTG, two distinct subpopulations were revealed via flow cytometry, providing an appropriate test case for the subsequent establishment of the RNA-Seq workflow (depicted in Fig. 2).

### Establishment of a reference data set

**Figure 3:**
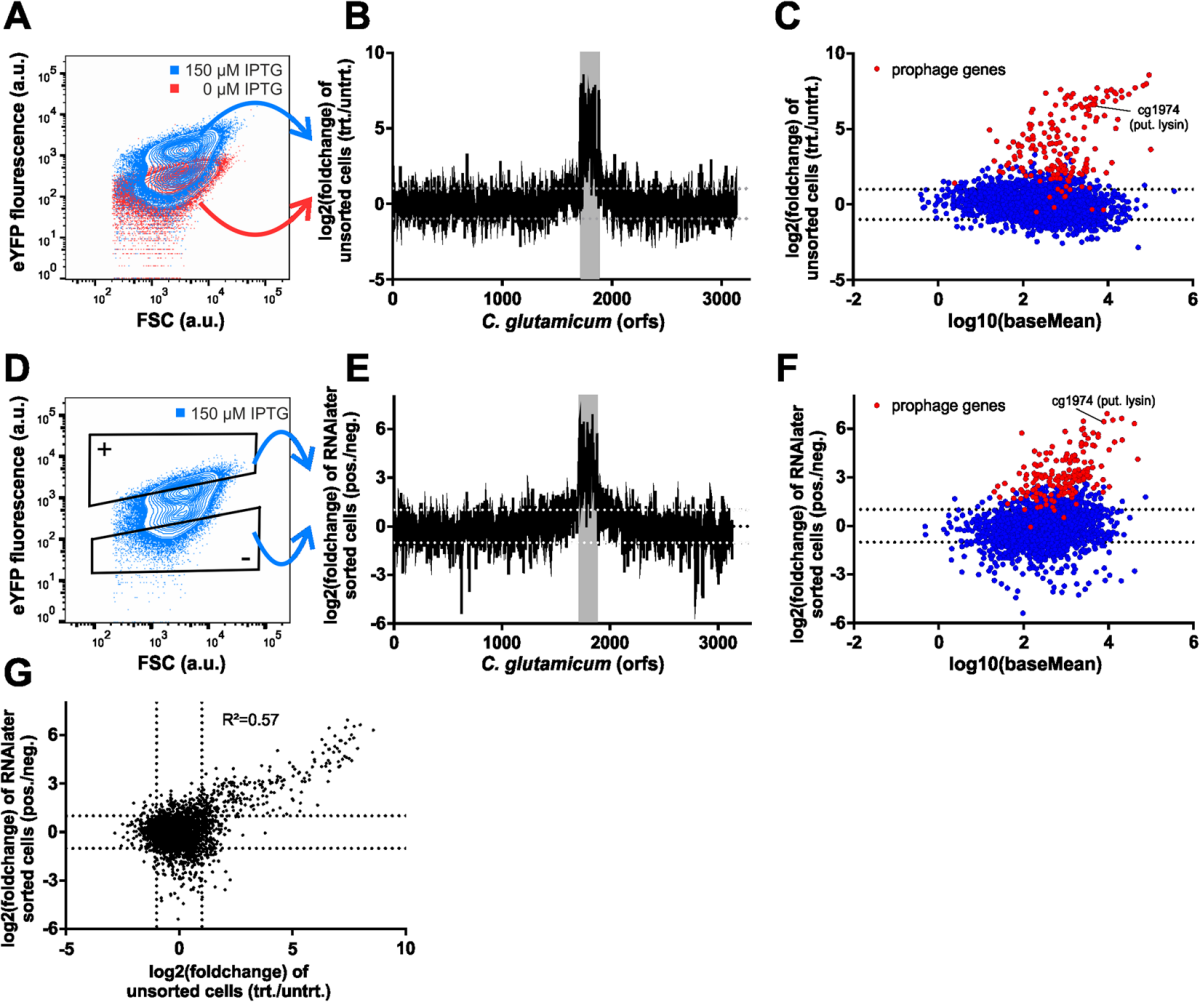
Differential gene expression analysis of prophage induction in *C. glutamicum*. *C. glutamicum*::*Plys-eyfp*/pAN6_*N-cgpS* cells were cultivated in CGXII medium with 2% (w/v) glucose and either with or without 150 µM IPTG. (**A**) Flow cytometry data show the activation of the phage reporter (blue), compared to the uninduced control (red). (**B,C)** From both samples RNA was extracted (without cell sorting) and analysed by RNA-Sequencing. The Log2(foldchange) was plotted against the coding regions (orfs) of the *C. glutamicum* genome. (**D)** Fluorescence-activated cell sorting to separate induced from uninduced cells from a heterogeneous population. (**E,F)** Differential gene expression analysis of the induced versus the uninduced subpopulation (see also Table S2). (**G)** Comparison of expression values of the sorted samples versus the unsorted reference data set (R^2^ = Pearson correlation coefficient).

To provide a reference data set for the validation of the envisaged RNA-Seq workflow, we performed RNA sequencing from unsorted samples treated via the standard protocol (see Material and methods). To this end, *C. glutamicum*::P*lys-eyfp*/pAN6_*N-cgpS* cells were grown in CGXII medium with and without 150 µM IPTG. After 6 hours, the cells were harvested, the RNA was extracted, and the library was prepared as described in material and methods. As expected, production of the truncated CgpS protein induced by addition of 150 µM IPTG led to a strong upregulation of gene expression within the prophage region (cg1890-cg2071) (Fig. 3A–C, Table S2). Most of the CGP3-encoded genes showed a significant upregulation, while none displayed reduced gene expression compared to the uninduced sample. Further upregulated genes from this experiment mainly encoded proteins involved in DNA repair and/or recombination (*recF*, cg1837, *ruvA*, *ruvB*, *xerC*, cg2633; see Table S2).

### RNA-Seq of 10^6^ bacterial cells – Workflow optimization

The overall workflow of subpopulation RNA-Seq is depicted in Fig. 2. In general, reporter cells were analyzed via flow cytometry. For each sample, 10^6^ cells were sorted in tubes and subsequently concentrated on filter plates to remove the culture supernatant. As the overall sorting procedure took approximately 15 minutes, the RNA was stabilized by using different stabilization agents, as described below (see also Fig. S2). For each RNA sample, a RIN analysis was performed as quality control. Only samples with a RIN value above 7 were used for sequencing. We also assessed the impact of a further decrease in sample material and sorting time by processing only 10^5^ cells, as described above. However, RNA sequencing revealed a low signal-to-noise ratio, and consequently, we proceeded with using samples of 10^6^ cells for further analysis. In the following, we briefly depict the most critical steps for workflow optimization.

When cellular biomass is limiting, as it is for RNA sequencing of 10^6^ cells, one of the most critical steps is the efficient extraction of high-quality RNA. Here, different RNA extraction protocols and methods were tested. The best results were achieved when using a non-column-based method such as NucleoZOL extraction. To further optimize our protocol, cells were pre-treated with different lysozymes. To compare different protocols, approximately one million cells (unsorted sample) were incubated either with chicken egg lysozyme or mutanolysine from *Streptomyces globisporus*. In our hands, the best results were obtained when combining both enzymes, but the treatment with chicken egg lysozyme only already yielded RNA of satisfactory amount and quality (Fig. S3).

A second critical step was the stabilization of the RNA to prevent degradation during the sorting procedure. Therefore, we compared different commercially available stabilization agents (RNAlater, Ambion, and RNAprotect, Qiagen). As an alternative approach, cells were directly sorted into a metal rack, which was cooled to −80 °C to immediately freeze sorted cells (Fig. S2). This shock-freezing approach was previously described for *Saccharomyces cerevisiae* as a suitable way to stabilize flow cytometry samples for subsequent gene expression analysis^21^. However, this approach appeared not to be appropriate for RNA sequencing of prokaryotic cells, as the extracted RNA was of poor quality and quantity (data not shown). RNA obtained from samples stabilized by RNAlater or RNAprotect was of acceptable quality and was further analyzed by sequencing. However, it has to be noted that both stabilization agents have a significant impact on the fluorescence of bacterial cells by denaturating cellular proteins and thus cannot be applied prior to cell sorting (Fig. S4). This drawback was already discussed in earlier studies^22,23^. Addition of stabilization agents prior sorting had a significant impact on *C. glutamicum* fluorescence in our case, but it was applied to HeLa cells infected with GFP-labeled *Salmonella* cells^13^. Red fluorescent proteins seem to be more stable against these agents than GFP-derived fluorescent proteins such as YFP and Venus^22,23^. However, the maturation time of red fluorescent proteins is typically far longer than of GFP derivatives^24,25^. This has to be considered in the face of the particular scientific question, as the actual response to a stimulus on the level of gene expression may be missed when cell sorting is performed at the time of maximal reporter output. These aspects hamper the analysis of transient changes in gene expression by the reported approach and demand a smart experimental design and the right choice of reporter proteins or fluorescent dyes.

### RNA-Seq of 10^6^ bacterial cells – Analysis of prophage induction

In the following, we applied the established workflow for the analysis of prophage induction in *C. glutamicum*. For RNA sequencing of sorted subpopulations, the CGP3 prophage was induced in a subpopulation of *C. glutamicum via* expression of a truncated silencer variant (Fig. S1, as described above)^20^. For this purpose, *C. glutamicum*::P*lys-eyfp*/pAN6_*N-cgpS* cells were cultivated in CGXII medium containing 150 µM IPTG. After six hours the cells were analyzed by flow cytometry and subpopulations were separated via FACS followed by filter plate concentration, NucleoZol RNA extraction and library preparation. The applied gating strategy is depicted in Fig. 1C. As described above, the cells were sorted into RNAlater or RNAprotect solution and afterwards concentrated on a filter plate. When comparing the induced versus the uninduced subpopulation, the upregulation of the CGP3 region was very prominent for cells sorted from the upper gate (Fig. 3D–F). Using RNAlater for stabilization, the strength of induction was slightly reduced compared to the unsorted control (Fig. 3E). When RNAprotect was used, the fold change of CGP3 genes further decreased, reaching a maximal log_2_-fold change of approximately 3.3 compared to 8.5 in the unsorted control and 6.9 in RNAlater sorted cells (Table 1, Fig. S5). Overall, the expression pattern of the unsorted control (Fig. 3B,C) and the sorted subpopulations (Fig. 3E,F) showed a consistent result, except the high number of downregulated genes for the RNAlater treated samples (Fig. 3G). This effect was, however, not observed when cells were sorted into RNAprotect and thus can likely be attributed to the impact of the stabilization agent. Earlier studies have reported the effects of stabilization agents on gene expression of *Escherichia coli*^22^. Those authors compared RNAprotect from Qiagen and RNAlater from Ambion. They noted, that RNAprotect massively changed the gene expression profile (~10% of all genes), while RNAlater just affected a few genes (>0.1%). The strong impact of RNAprotect was attributed to the induction of the response of *E. coli* to acid stress.

**Table 1:** Strains and plasmids used in this study.

Strains or plasmids	Relevant characteristics	Source or reference
Strains		
*C. glutamicum* ATCC 13032	Biotin-auxotrophic wild type	^48^
*C. glutamicum*::*Plys-eyfp*	Derivative of ATCC 13032 containing the integrated prophage reporter P_*lys*_-*eyfp* into the intergenic region of cg1121-cg1122	^20^
*C. glutamicum* Δ*dtxR*	In-frame deletion of the *dtxR* gene	^37^
*C. glutamicum ΔdtxR*::*Plys-eyfp*	Derivative of the Δ*dtxR* strain containing the integrated prophage reporter P_*lys*_-*eyfp*	This study
*C. glutamicum ΔrecA*::*Plys-eyfp*	In-frame deletion of *recA*, containing the integrated prophage reporter Plys-eyfp	^18^
*E. coli* DH5α	*supE*44 *ΔlacU*169 (*ϕ80lacZ*DM15) *hsdR*17 *recA*1 *endA*1 *gyrA*96 *thi-*1 *relA*1	Invitrogen
Plasmids		
pAN6	*Kan*^*R*^*; C. glutamicum*/*E. coli* shuttle vector for gene expression under control of the *tac* promoter; (P*tac*, *lacI*^*q*^*, pBL1 ori*V_*C.g*._, pUC18 *ori*V*E.c*.)	^17^
pAN6-*N*-*cgpS*	Kan^R^; pAN6 derivative containing the first 65 amino acids of the *cgpS* gene	^20^
pAN6-*dtxR*	*Kan*^R^; variant of pAN6. Contains *dtxR* gene under *tac* promoter for complementation studies.	This study
pAN6-*recA*	*Kan*^R^; variant of pAN6. The *recA* gene was cloned under the influence of the tac promoter (for complementation).	This study
pJC1	Kan^R^; *E. coli*/*C. glutamicum* shuttle vector (pHM1519 *ori*_*Cg*_, pACYC177 *ori*_*Ec*_)	^49^
pJC1-*venus*-term	Kan^R^; pJC1 derivative containing the *venus* gene and additional terminators	^50^
pJC1-*PdivS-venus*	Kan^R^; pJC1 derivative containing the *venus* gene under the *divS* promoter control	This study
pK18*mobsacB*-cg1121/1122-P_*lys*_-*eyfp*	Plasmid for integration of the *lysin* promoter (of cg1974) fused to *eyfp* into the intergenic region of cg1121-cg1122	^18^

Remarkably, CGP3 flanking regions (approximately cg1619-cg1890, left flank, and cg2071-cg2311, right flank) showed a significant upregulation in all experiments (fold change >2), which was especially evident in the analysis of sorted subpopulations treated with RNAlater (Table S1, Fig. 3E). This effect was, in fact, attributed to regional replication at the CGP3 locus and is further discussed in the next paragraph.

In addition to the CGP3 prophage and its flanking regions, several further genes displayed a significantly altered expression level when comparing prophage induced versus uninduced subpopulations. However, when focusing on genes showing a similar trend in at least two out of the three comparisons (Table S2, Figs 3 and S5) only a small number of genes became evident. Next to the CGP3 region, a putative pseudogene (cg1508) of the prophage CGP1 was co-induced in the prophage-induced subpopulation, indicating a regulatory link between the two cryptic phages. The prophage silencer CgpS also binds to regions within the CGP1 element^20^. Thus, interference with CgpS activity may have caused this effect in our experiment.

Furthermore, a putative operon close to the origin of replication (cg0004-0006) showed a significant upregulation in the prophage-induced subpopulation. This operon includes the genes encoding for the DNA polymerase β subunit (*dnaN*), the recombination protein RecF and a Zn-ribbon containing putative RNA binding protein (cg0006), which is conserved in actinobacteria. These findings suggest that the CGP3 prophage recruits parts of the replication/recombination machinery of the host for efficient phage DNA replication.

### Regional replication at the CGP3 locus upon prophage induction

**Figure 4:**
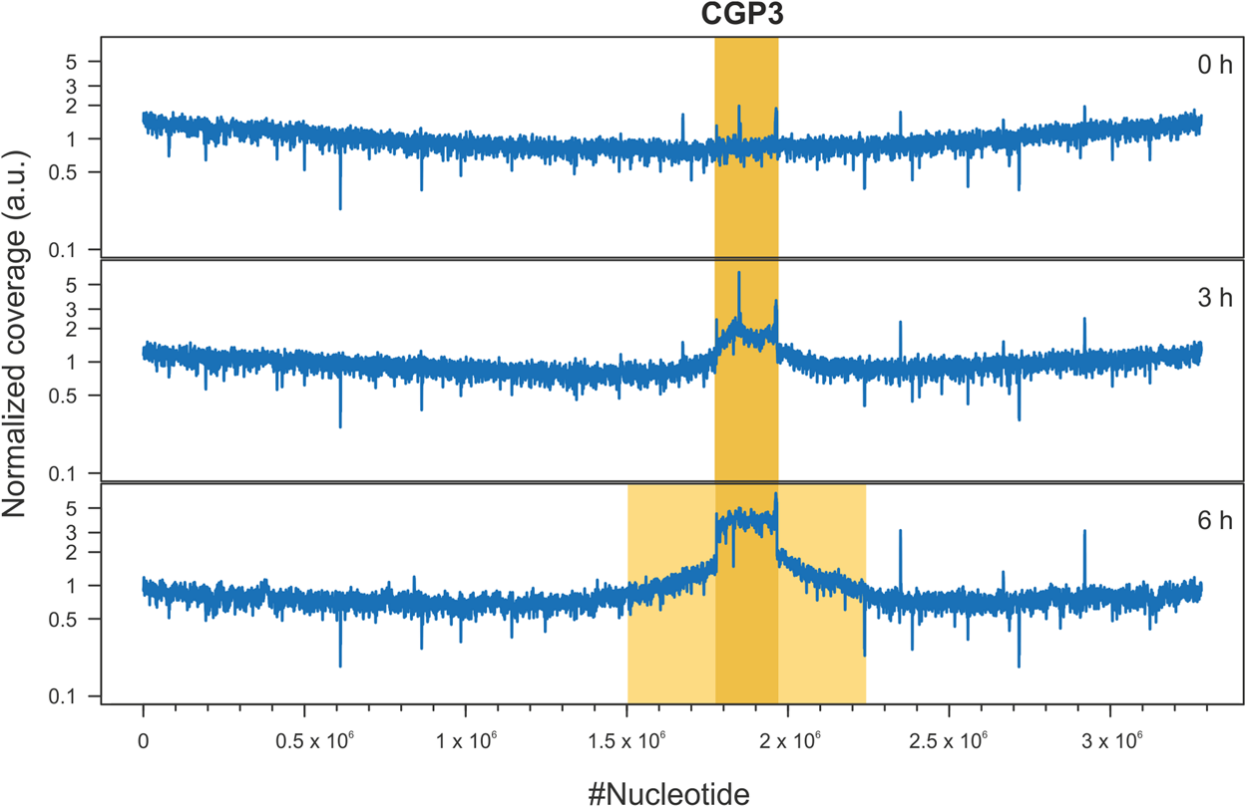
Genome re-sequencing reveals regional replication at the CGP3 locus. *C. glutamicum*::*Plys-eyfp*/pAN6_*N-cgpS* cells were cultivated in the presence of 150 µM IPTG to induce *N-cgpS* expression triggering CGP3 prophage induction. Samples were taken at different time points (0, 3 and 6 hours), the DNA was extracted and sequenced. Plots show the respective mean normalized genomic coverage on a logarithmic scale against the respective genomic position. The CGP3 locus is highlighted in orange; flanking regions affected by regional replication are shaded in light orange.

Remarkably, the RNA sequencing of CGP3-induced cells (sorted and unsorted samples) revealed an increased coverage of genomic regions flanking the CGP3 *attL* and *attR* sites (Fig. 3B,E). We assumed that this effect might be the result of regional replication at the CGP3 locus upon prophage induction. To assess whether this finding coincided with an increased coverage of the genomic DNA of the CGP3 flanking regions, we profiled the effect of prophage induction by whole-genome sequencing of samples harvested at different time points after CGP3 induction (Fig. 4). To this end, *C. glutamicum*::*Plys-eyfp*/pAN6_*N-cpgS* cells were induced with 150 µM IPTG. Prior to induction (0 hours), the highest genomic coverage was observed at the origin of replication, decreasing towards the terminator site. This is the typical pattern of multi-fork replicating bacterial cells. Induction of CGP3 resulted in a decreased coverage at the origin, reflecting the growth arrest as a consequence of prophage induction. After 3 hours, a distinct peak arose at the CGP3 locus as a result of phage DNA excision and replication (Fig. 4). In line with the RNA sequencing data, genome sequencing confirmed the increased coverage of the CGP3 flanking regions (approximately cg1619-cg1890, left flank, and cg2071-cg2311, right flank), emphasizing regional replication of CGP3 upon induction. This replication is supposed to start within the still genomically integrated prophage and progresses across the phage *attL* and *attR* attachment sites, leading to an increased genomic coverage in this area.

Similar effects have been observed for a number of different phages, including different *E. coli* phages^26^, *Bartonella grahamii*^27^ and for phages of *Salmonella enterica*^28^. However, the first report of regional replication (or ‘escape replication’) goes back to 1967. Fukasawa and coworkers published a series of reports analyzing the upregulation of the galactose operon during phage induction^29^. While the precise molecular mechanism remains unclear, they showed, in their first study, that this effect depends on the DNA synthesis of the host bacteria. The galactose operon is located next to the prophage region and in following studies it was shown, that regional replication at the prophage element leads to an increased copy number of flanking regions as well as causing an elevated expression of respective genes in this genomic area^30^. The molecular reason of the regional replication was further studied and was linked to the function of the integrase and excisionase of the phage λ^31–33^. The phenomenon of regional replication or escape replication was also recently described for prophages of *S. enterica*^28^. In this study, the authors could show that the knockout of genes encoding the integrase and the excisionase will lead to regional replication. The effect of regional replication may be explained by a delayed excision of the prophage from the genome, but the molecular and physiological reasons still remain obscure and are a focus of further studies. It is, however, an educated guess that the initiation of regional replication prior to excision and – along with it – the upregulation of adjacent genes is beneficial for the phage and likely contributes to efficient viral proliferation.

### Iron-triggered prophage induction in early exponential phase cultures

In previous studies, a mutant of the gene encoding the global iron regulator DtxR displayed a significantly increased fraction of CGP3 induced cells^17^. Since DtxR functions as a repressor of genes involved in iron uptake, we hypothesized that increased intracellular Fe^2+^ levels triggered cellular stress responses (e.g., the SOS response) *via* the Fenton reaction releasing hydroxyl radicals. The SOS response, induced by DNA damage, may also lead to prophage induction *via* various mechanisms. Previous studies have demonstrated a causative relationship of the *C. glutamicum* SOS response and the induction of the large cryptic prophage CGP3^18,19^.

**Figure 5:**
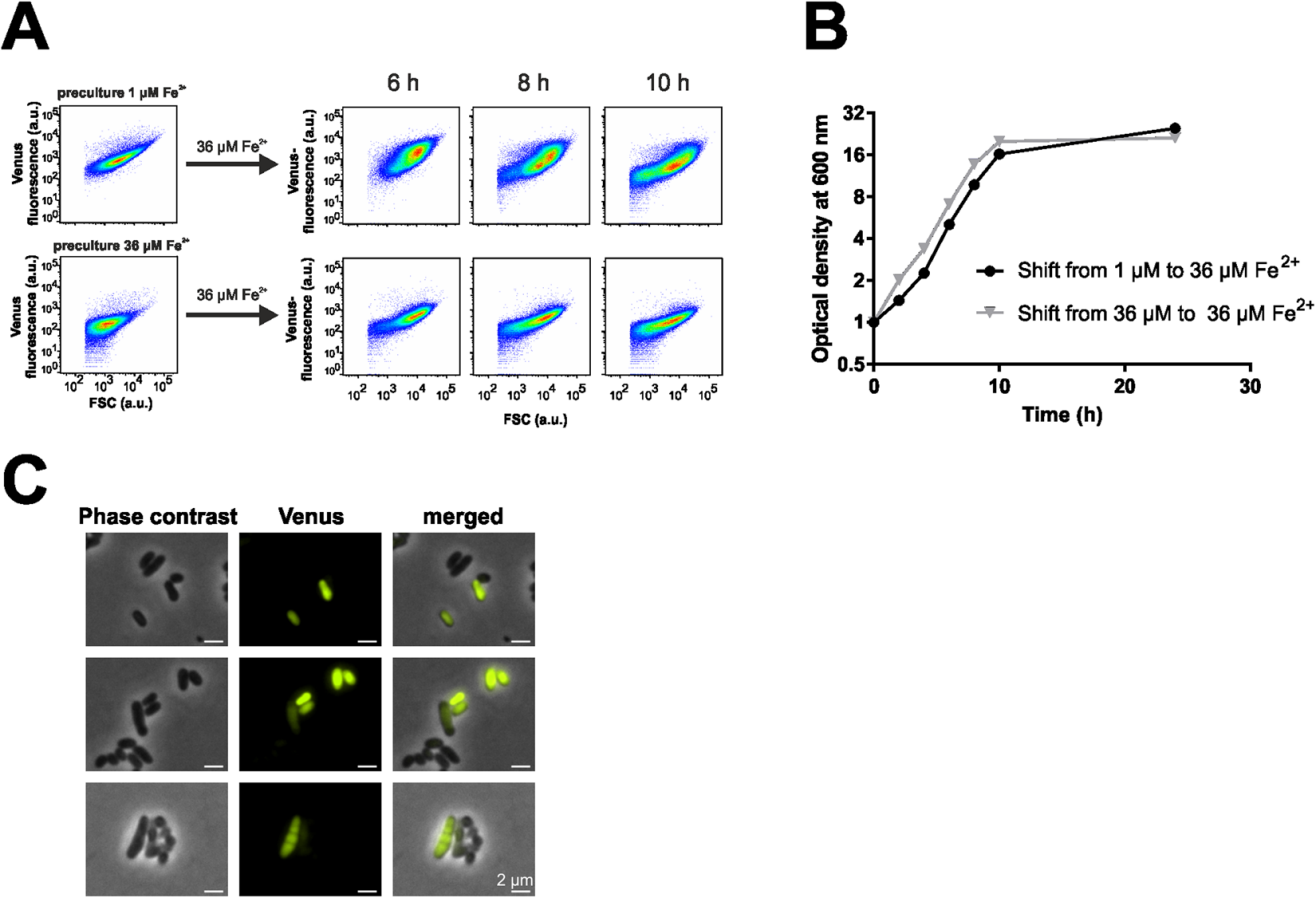
Activation of the SOS response in *C. glutamicum* lag phase cultures triggered by iron fluctuations. *C. glutamicum*/pJC1_*PdivS-venus* cells were cultivated in CGXII medium with 2% (w/v) glucose under iron limitation (1 µM FeSO_4_) and under conditions of sufficient iron supply (36 µM FeSO_4_). (**A**) Stationary phase cells from the preculture (either 1 or 36 µM FeSO_4_) were transferred into fresh CGXII medium with 36 µM FeSO_4_ and analysed by flow cytometry. (**B**) Growth of the strains after the transfer into fresh medium. (**C**) Microscopy images of cells upon iron upshift confirmed a heterogeneous reporter output.

**Figure 6:**
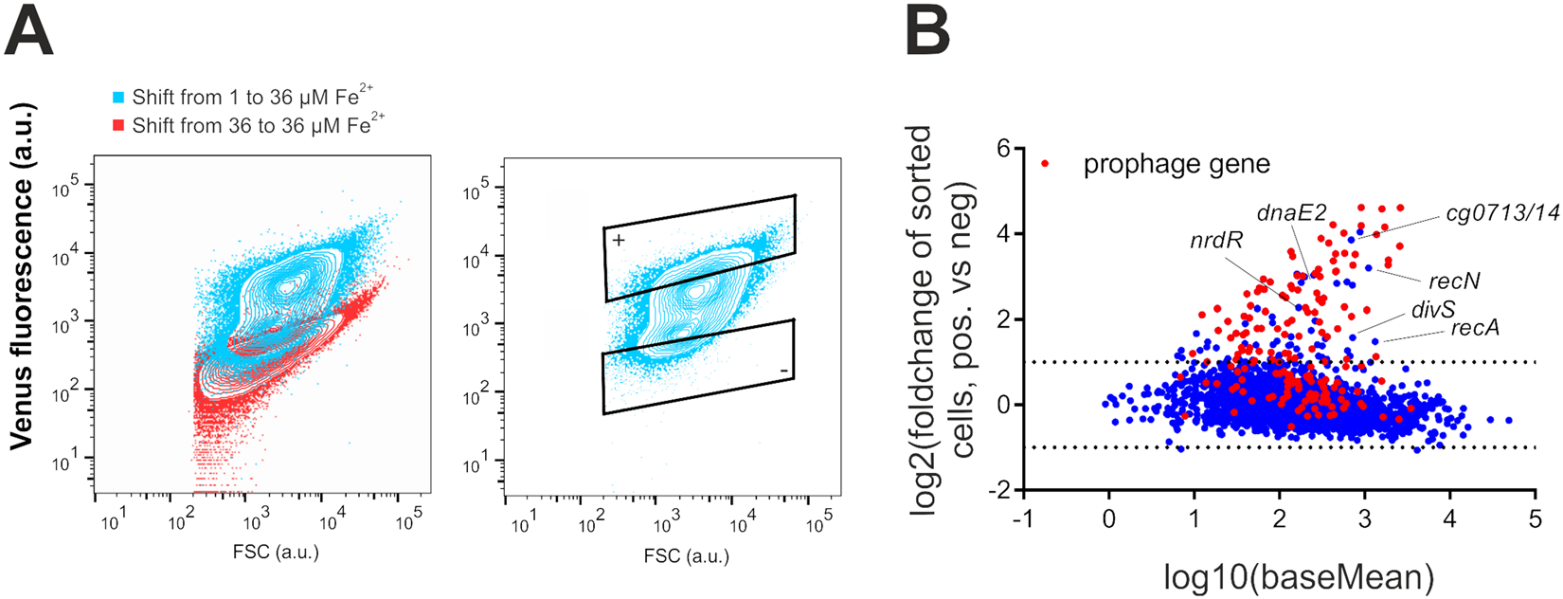
Iron triggered prophage induction in *C. glutamicum*. Shown is a differential gene expression analysis of subpopulations isolated from an iron upshift experiment. *C. glutamicum*/pJC1_*PdviS-venus* cells were first cultivated in CGXII medium with 1 µM iron and then shifted to medium with 36 µM iron. (**A)** Based on the output of the *divS* promoter fusion, 10^6^ cells of each subpopulation were sorted and treated according to the established RNA-Seq workflow. (**B)** Differential gene expression analysis: As expected, several SOS genes showed an upregulation in the *divS*^+^ subpopulation. Remarkably, also the expression of genes located within the CGP3 prophage element (red dots) were significantly increased indicating iron-triggered prophage induction.

To further validate our RNA-Seq workflow, we investigated the impact of fluctuating iron concentrations on the cellular SOS response by using a *divS*^34^ promoter fusion (cg2113, SOS-inducible cell-division suppressor) to *venus*^19^. To this means, *C. glutamicum* cells containing the pJC1_*PdivS-venus* plasmid were cultivated in 1 µM FeSO_4_ containing CGXII medium with 2% glucose. Iron-starved cells were subsequently transferred to fresh CGXII medium containing 36 µM FeSO_4_ (standard conditions). As a reference sample, cells grown under a non-limiting iron supply (36 µM FeSO_4_) were transferred to fresh medium with the same initial iron concentration. The growth and the fluorescence signal were measured over time using flow cytometry (Fig. 5). Whereas cells shifted from iron starvation were delayed in the initial growth phase, cells that had not suffered iron limitation immediately continued exponential growth. This observation is in line with the flow cytometry analysis of the reporter strain. Here, the transfer of iron-starved cells into fresh medium caused an increased *divS* promoter activity in a significant fraction of cells, reflecting an induction of the SOS response. The most prominent split of the reporter output was observable after 8 hours of cultivation, a delay presumably due to the maturation of the reporter protein (Figs 5A and S6). However, this population heterogeneity only occurred when protocatechuic acid was added to the medium (Fig. S6), which functions as an iron chelator conferring fast uptake of iron in the early exponential phase. Further microscopic analysis of cells exposed to a shift from low to high iron conditions also confirmed significant cell-to-cell variability with respect to the *divS* reporter output (Fig. 6D). These findings are in line with our hypothesis that fluctuations in extracellular iron availability (low → high iron) lead to an induction of the cellular SOS response in the early exponential growth phase.

In the following, this effect was further studied by applying the established RNA-Seq workflow to *divS*-positive and -negative subpopulations (Table S3, Figs 5 and 6). This transcriptomic approach revealed a significant upregulation of prophage genes (cg1890-cg2071, Table S3 and Fig. 6) in the *divS*-positive subpopulation, indicating that a shift to high iron concentrations led to an SOS-dependent prophage induction in early-exponential cells.

In addition to the phage genes, almost all other genes (e.g., cg0713, cg0714, *dnaE2*, cg1318, *recN*) showing an upregulation in the *divS*^+^ subpopulation are targets of the SOS repressor LexA^35^, confirming the activation of this DNA damage response triggered by fluctuating iron levels. Furthermore, cg0053, encoding the high-affinity iron uptake system, was more highly expressed in the *divS*^+^ subpopulation (Table S3). This finding indeed suggested that a higher iron uptake rate may have caused SOS-dependent prophage induction in this subpopulation.

**Figure 7:**
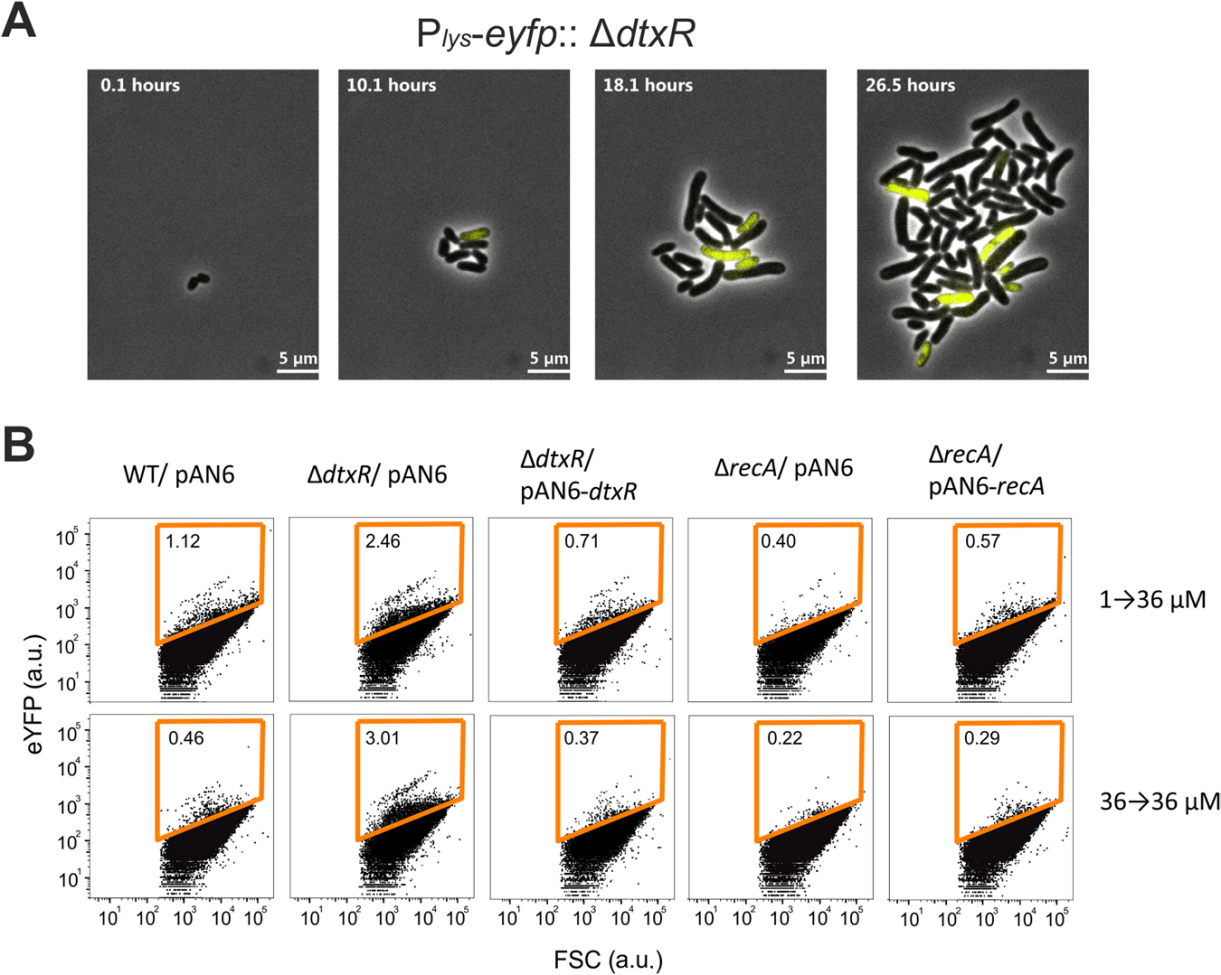
Iron-triggered prophage activation is dependent on the cellular SOS response. (**A**) Time lapse studies of a Δ*dtxR* strain containing the genomically integrated prophage reporter P_*lys*_-*eyfp* (strain: *C. glutamicum* Δ*dtxR*::P_*lys*_-e*yfp*). Cells were grown in a microfluidic chip device (see material and methods) in CGXII minimal medium with 2% (w/v) glucose and under a constant flow rate of 300 nl min^−1^. (**B**) *C. glutamicum* wild type, Δ*dtxR* and Δ*recA* strains, containing the integrated P_*lys*_-*eyfp* reporter, were cultivated under iron up-shift conditions (1 → 36 µM FeSO_4_). After 4 h samples were taken and analyzed by flow cytometry. Whereas a prophage-induced subpopulation is clearly visible in Wt and ΔdtxR cells (1.28% and 4.61%, respectively), almost no activity of the phage reporter was observed in Δ*recA* cells. Deletion of *dtxR* and *recA* was complemented by transforming the respective strains with the plasmids pAN6-*dtxR* or pAN6-*recA*, respectively. Shown are representative scatter plots from three biological replicates; absolute values varied between replicates whereas the overall trend was consistent.

**Figure 8:**
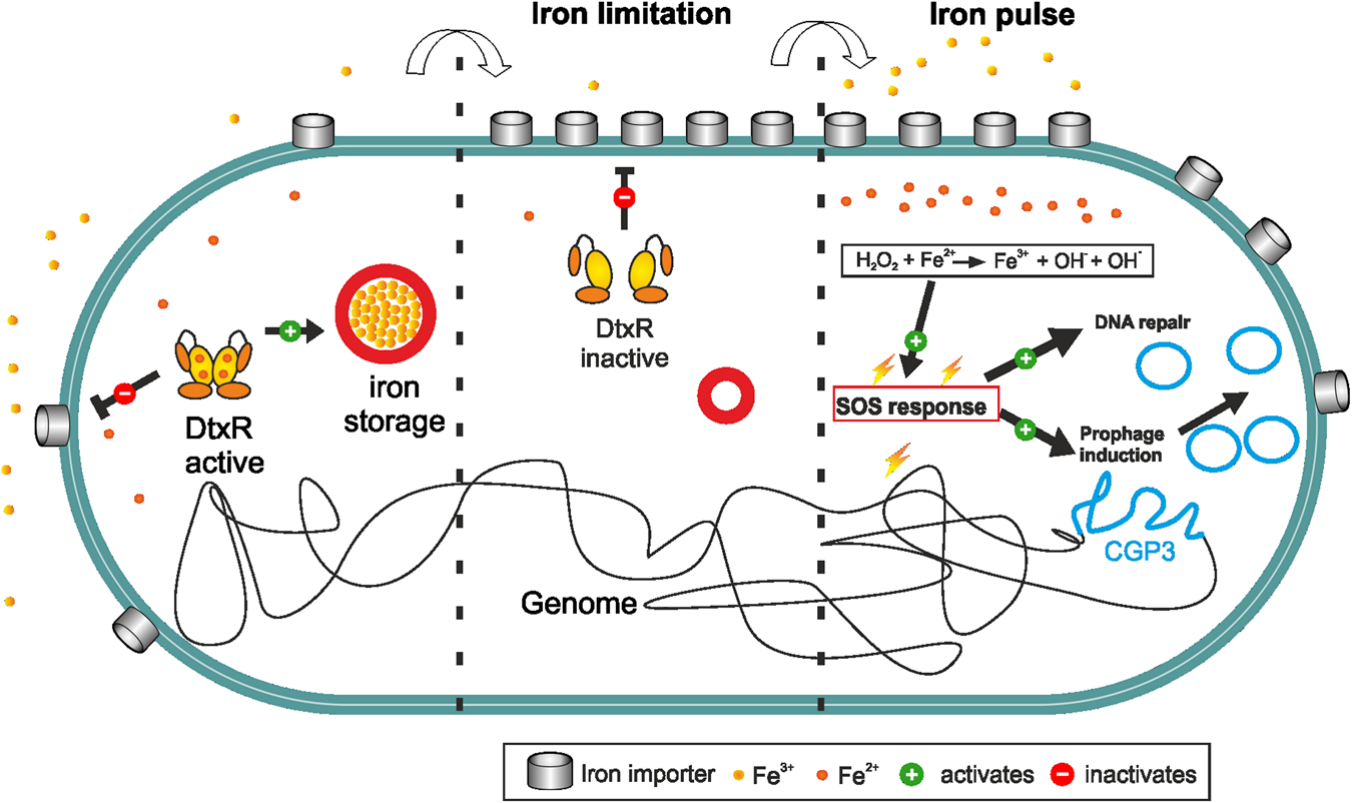
Model of iron-triggered prophage induction. Under conditions of sufficient iron supply, the global iron regulator DtxR is in its (Fe^2+^-bound) active state and represses genes encoding high-affinity iron uptake systems while activating expression of ferritin^37^. Under iron limiting conditions, DtxR dissociates from its target promoters and iron uptake is highly upregulated. A fast transition to medium containing high iron concentrations (e.g. 36 μM) will now lead to fast uptake of iron. Increased intracellular Fe^2+^ levels may cause oxidative stress and lead to SOS-triggered prophage induction in a fraction of cells (Figs 6 and 7).

Iron is a key element for bacterial species. It is an essential cofactor for numerous enzymes, but elevated levels may cause cellular damage by the reactivity of hydroxyl radicals produced by the Fenton reaction or induced by iron-oxygen complexes^36^. An impact of iron on prophage induction was previously postulated, as an increase in phage gene expression was observed in a mutant lacking the global iron regulator DtxR^17,37^. In this study, we further confirmed the higher phage activity in the Δ*dtxR* mutant: Under standard conditions, a Δ*dtxR* mutant carrying the phage reporter exhibited an elongated cell morphology, as typical for SOS-induced cells, and a significantly increased phage reporter output in approximately 5% of the cells (Fig. 7A). In contrast, prophage induction was hindered in a SOS-defective mutant lacking the *recA* gene (Fig. 7B). These phenotypes were complemented by basal expression of *dtxR* and *recA* from P_*tac*_, respectively. Altogether, these findings corroborate our hypothesis that prophage induction in the early exponential phase is a consequence of an intensive iron uptake, leading to an induction of the cellular DNA damage response (Fig. 8). In line with our findings, a transcriptome study of *Salmonella enterica* serovar Typhimurium revealed that lag-phase cultures transiently accumulate iron leading to oxidative stress^38^. Iron also triggers induction of the prophage λSo in *Shewanella oneidensis* and thereby promotes the release of extracellular DNA as an important matrix component for biofilm formation^39^. Taken together, these findings illustrate how microorganisms may even harness the toxic effects of intracellular iron accumulation to adapt to a particular ecological niche, such as by supporting biofilm formation.

## Conclusions

Here, we present a novel workflow for the transcriptome analysis of bacterial subpopulations. The impact of different methodological approaches was compared to achieve optimal mRNA stabilization during cell sorting. While significant influences on the expression profile were observed in the different experiments, the overall approach was successfully applied to study prophage induction in a subpopulation of *C. glutamicum*. For the first time, RNA sequencing of prophage-induced subpopulations revealed regional replication at the CGP3 locus – a phenomenon so far obscured by bulk approaches. Finally, we applied the presented workflow for the analysis of iron-triggered prophage induction in the early exponential phase of *C. glutamicum* cultures. These examples nicely demonstrate the potential of the presented workflow to unravel phenotypic dynamics of bacterial populations, which may reflect crucial adaptive traits of a particular species or microbial community.

## Material and Methods

### Bacterial strains and growth conditions

The bacterial strains and plasmids used in this study are listed in Table 1. *E. coli* DH5α was used for cloning procedures and was therefore cultivated in lysogeny broth (LB) medium or agar plates at 37 °C^40^. *C. glutamicum* ATCC 13032 was used as the wild-type strain^41^. For growth measurements or fluorescence-activated cell sorting (FACS) a single colony was picked from an agar plate and cultivated in brain heart infusion (BHI) medium (DifcoTM BHI, BD, Heidelberg, Germany) medium at 30 °C for 6 to 8 hours. Subsequently, this pre-culture was centrifuged, and the resulting pellet was used to inoculate an overnight culture in CGXII^42^ minimal medium containing 2% (w/v) glucose and 30 mg·l^−1^ protocatechuic acid. Finally, the overnight culture was used to inoculate fresh CGXII medium to an OD_600_ of 1. If necessary, 50 mg·ml^−1^ (*E. coli*) or 25 mg·ml^−1^ (*C. glutamicum*) kanamycin was added.

### Recombinant DNA work

Oligonucleotides and plasmids used in this study are listed in Table S1. Sequencing of plasmids and synthesis of oligonucleotides were conducted by Eurofins MWG Operon (Ebersfeld, Germany). The standard methods of PCR and restriction were performed according to established protocols^40^. Gibson assembly^43^ was routinely applied for plasmid construction.

### Cultivation in microfluidic chip devices

For the cultivation of single cells, an in-house-developed microfluidic platform was used as described in previous studies^44,45^. Imaging in the fluorescence and phase-contrast channels was performed at an 8 min interval using an inverted epifluorescence microscope (TI-Eclipse, Nikon GmbH, Düsseldorf, Germany). A constant medium flow of 300 nl·min^−1^ was adjusted to ensure stable environmental conditions. Cells were trapped in the growth chamber; a constant temperature of 30 °C was set using the incubator system of PeCon GmbH (Erbach, Germany).

### Fluorescence microscopy

Images of fluorescent samples were taken on an AxioImagerM2 (Zeiss, Oberkochen, Germany) equipped with a Zeiss AxioCam MRm camera and an EC Plan-Neofluar 100×/1.3 Oil Ph3 objective. The filter set 46 HE YFP was used to detect fluorescence. The software AxioVision version 4.8 was used to acquire fluorescent images. To this end, *C. glutamicum* cells were cultivated as described in the section ‘Bacterial strains and growth conditions’. For microscopy of cell samples, a microscopic slide was coated with a thin 1% (w/v) agarose layer based on Tris-acetate-EDTA buffer. Subsequently, 5 µl of the culture was pipetted on this layer and analyzed.

### Flow cytometry and fluorescence-activated cell sorting (FACS)

For flow cytometry analysis, *C. glutamicum* cells were diluted to an OD_600_ of 0.1 in cold PBS-buffer (137 mM NaCl, 2.7 mM KCl, 10 mM Na_2_HPO_4_, 1.8 mM KH_2_PO_4_). A FACS Aria II flow cytometer (Becton Dickinson, Son Jose, USA) equipped with a 488 nm blue solid laser was used for analysis and cell sorting^46^. Forward-scatter characteristics (FSC) resulted from the small-angle scatter, while side-scatter characteristics (SSC) were recorded as orthogonal scatter of the 488-nm laser. Fluorescence (eYFP/Venus) was detected by a 502-nm long-pass and a 530/30 nm band-pass filter set. The FACS software DIVA 6.0 was used for the recording. Thresholding on the FSC/SSC was applied for all measurements and removed for the sorting procedure. Cell sorting was achieved by pre-gating the cells twice. The first gate was set around the FSC height and width signal to remove cell doublets. The next gate was set around the FSC height and fluorescence height signal to remove non-cell particles. From these pre-gating settings, the cells were sorted with the four-way purity mask and a threshold rate of 8000 events/s. Cells were sorted in 5 ml Eppendorf tubes. To this end, a new plastic tube holder was manufactured in house sharing the same shape of the 15 ml tube rack, but just the upper 2 cm and leaving out the bottom part (Fig. S1). The tubes were filled with 2 ml RNAlater (Ambion ordered by Thermo Fisher Scientific) or RNAprotect (Qiagen) solution and a small magnet. Below the tubes, a magnetic stirrer was installed to properly mix the solution during sorting. Another manufactured tube holder of the same shape as the 15 ml original tube rack was built from aluminum. This tube holder was cooled down to −80 °C prior sorting, to freeze the cells right after they were sorted. After sorting, the cells were concentrated on a MultiScreen HTS filterplate (Millipore, Billerica, USA) to remove the sheath fluid and RNA stabilization agents. Subsequently, the filter was cut from the plate and frozen in liquid nitrogen for later use.

### RNA extraction and quality control

RNA was extracted using two different methods, depending on the sample size. For the extraction of RNA from large volumes, a modified protocol of the RNeasy Kit (Qiagen, Hilden, Germany) was applied. To this end, 20 ml of the bacterial culture were transferred in a 50 ml Falcon tube filled with ice. The tube was immediately centrifuged for 10 minutes at 4500 rpm at 4 °C. The supernatant was discarded, and the pellet was frozen in liquid nitrogen. Subsequently, the pellet was stored at −80 °C. For RNA extraction, the pellet was dissolved in 350 µL RLT buffer (Qiagen, Hilden, Germany) containing 1% (v/v) 1 M dithiothreitol (DTT) and transferred into a 2 ml tube. Then, 250 mg glass beads (µM) were added, and the tube was placed into a CapMix (3 M ESPE AG, Seefeld) for 1 × 15 s and 1 × 30 seconds. Afterwards, the sample was centrifuged for 2 minutes at maximum speed. The supernatant was transferred into a new tube and mixed with 250 µl of pure ethanol (−20 °C). The solution was transferred to the spin column and centrifuged. The column was washed with 350 µl RW1-buffer. For the on-column DNA digestion, the RNase-Free DNase Set (Qiagen, Hilden, Germany) was used. The column was incubated with 80 µl DNase Mix (70 µl RDD buffer +10 µl DNase I (4 units/µl)) for 15 minutes. The column was then washed with 350 µl RW1 buffer, followed by two washing steps with 500 µl RPE buffer. The RNA was eluted with two times 40 µl RNase-free water.

Extraction of RNA from small samples volumes (e.g., 10^6^ cells) was conducted with NucleoZol (Macherey-Nagel, Düren, Germany). To this end, the frozen filter plate containing one million cells was pre-treated with cell wall-degrading enzymes. The filter plate was incubated at room temperature in 100 µl TE buffer (10 mM Tris-HCl, 1 mM EDTA, pH 7.5) containing 10 µl lysozyme (Carl Roth, Karlsruhe, Germany) from chicken egg (25 mg/ml) and 5 µl mutanolysine (Sigma Aldrich, Taufkirchen, Germany) from *S. globisporus* ATCC 21553 (50 U/µl). The incubation time was reduced from 15 minutes to 5 minutes for later studies, which showed only a minor influence on the extraction yield. Subsequently, 300 µl NucleoZol was added to the sample and mixed. Then, 100 mg glass beads were added, and the tube was shaken for 30 seconds in the CapMix. Next, 100 µl nuclease-free water was added, and the tube was shaken again for 15 seconds. The solution was incubated for 15 minutes and then centrifuged for 30 minutes at 12,000 × *g* and 4 °C. The upper phase was separated from the lower blue phase. To the upper phase, 5 µl 4-bromanisole (Sigma-Aldrich, Taufkirchen, Germany) was added and mixed. After 5 minutes’ incubation, the tube was centrifuged again for 15 minutes at 4 °C. The aqueous upper phase was carefully transferred into a new tube, and 2 µl glycogen (20 mg/ml) (Sigma) together with 1 volume of isopropanol (−20 °C) was added and mixed vigorously. The tube was placed at −20 °C for 1 hour and afterwards centrifuged for 60 minutes at 4 °C. The pellet was washed twice with 500 µl ice-cold 75% ethanol (v/v) and was centrifuged for 5 minutes at 4 °C. The residual ethanol was evaporated, and the RNA pellet was resuspended in 15 µl nuclease-free water. The total RNA solution was stored at −80 °C. DNase treatment was applied directly before rRNA depletion. To this end, 2 µl of 10 × DNase buffer (100 mM Tris HCl, 25 mM MgCl_2_ and 5 mM CaCl_2_, pH 7.6) and 1 µl of DNase from the RNase-Free DNase Set (Qiagen, Hilden, Germany) were incubated for 10 minutes with the total RNA sample. This mixture was directly used for the rRNA depletion.

RNA quality was assessed using the Agilent Tape station 2200 (Agilent Technologies, Waldbronn, Germany) with the High Sensitivity RNA ScreenTapes according the manufacturers’ instructions. Successful removal of DNA was tested by standard PCR with the primers fw_PgntK_OL (cg2732) and rv_PgntK_OL (cg2732).

### rRNA depletion (Ribo-Zero)

The Ribo-Zero rRNA Removal Kit (Gram-Positive Bacteria) (Epicentre/Illumina, Munich, Germany) was used to remove rRNA of all samples. Samples received from large volumes were treated according to the manufacturer’s instructions. Here, 1 µg DNase-I-treated total RNA was used as input. The rRNA-depleted RNA was recovered with ethanol for 2 h at −20 °C. The pellet was resolved in 5 µl nuclease free water.

For smaller samples sizes (e.g., 10^6^ cells) a modified protocol was used. The “Protocol for Removal of rRNA from Small Amounts of Total RNA” from Clontech was used since it is recommended for RNA amounts in the range from 2–100 ng. Here 16 µl from the DNase treated RNA were mixed with 2 µl Ribo-Zero reaction buffer and 2 µl Ribo-Zero removal solution, following the protocol. Finally, the RNA was precipitated by the addition of ethanol again, as described above. The pellet was resuspended in 5 µl nuclease-free water.

### cDNA library, qPCR and RNA-sequencing

Sequencing libraries were generated using the TruSeq Stranded emRNA Library Prep Kit (Illumina, Munich, Germany). In all cases, the mRNA selection was omitted, since we already depleted rRNA and bacterial RNAs do not have mRNA with adenylated ends. The starting point was the fragmentation of the DNase-I-rRNA-depleted RNA. The 5 µl from the rRNA depletion was mixed with 14.5 µl of the Fragment, Prime, Finish Mix, and the solution was incubated for 6 minutes at 94 °C. The steps of first-strand cDNA synthesis, second strand cDNA synthesis and 3′ prime adenylation were carried out following the manufacturer’s instructions. For the adapter ligation, the amount of used adapters was changed for the samples of 1 million cells. Here, only 1.5 µl of RNA Index Adapters was used, and 1 µl resuspension buffer was added. PCR enrichment was performed with up to 14 cycles for RNA received from 1 million cells and with 8 cycles for the received from large volumes.

To quantify the final cDNA library of each sample, the KAPA library quantification Kit for Illumina platforms from KapaBiosystems (Roche, Unterhaching, Germany) containing the Universal qPCR MasterMix and the DNA Illumina standards 1–6 was used following the manufacturer’s instructions. Oligonucleotides were obtained from Eurofins MWG Operon (Ebersfeld, Germany). The qPCR itself was conducted on the qTOWER 2.2 (Analytic Jena, Jena, Germany).

All RNA-Seq experiments were paired-end (2 × 76 cycles) and were conducted on a MiSeq platform (Illumina, Munich, Germany) using the MiSeq Reagent Kit v3 (150-cycle). The input RNA was fragmented before cDNA synthesis to an average length of 150 bp. Four cDNA samples were pooled in one sequencing run in approximately equimolar amounts.

### Extraction of genomic DNA

DNA was extracted using the NucleoSpin Microbial DNA Kit (Macherey-Nagel, Düren, Germany) and the NucleoSpin Bead Tubes Type B following the manufacturer’s instructions. To this end, 1 ml culture concentrated to an OD_600_ of 10 was used to extract genomic DNA. Samples were added to tubes filled with glass beads and homogenized using the Precellys 24 (VWR, Ismaning, Germany) with 3 pulses of 20 seconds at 4500 rpm. The DNA was resuspended in nuclease-free water.

### DNA library and DNA sequencing

DNA libraries were generated using the TruSeq DNA PCR-Free LT Library Prep Kit from Illumina. In a first step, 4 µg DNA dissolved in 100 µl resuspension buffer was size-fragmented with 5 cycles of 15 seconds sonication (breaks of 90 seconds) at 4 °C in the Bioruptor Pico (Diagenode s.a., Liege, Belgium).

### Read processing and mapping

RNA and DNA sequencing results were gained as Illumina sequencing reads in FASTQ format. These reads were further processed with trimmomatic version 0.36 (Bolger, Lohse, and Usadel 2014). This tool removes Illumina adapters and trims low-quality bases. Remaining reads were aligned against the *Corynebacterium glutamicum* ATCC 13032 reference genome (GenBank accession number BX927147.1)^41^ with the bowtie2 aligner tool version 2.3.2^47^ allowing 1 base mismatch and using the end-to-end alignment mode. Aligned data files were further converted with SAMtools version 1.3.1 (Li *et al*. 2009) to create sorted bam files for differential gene expression analysis or to create read coverage statistics (samtools depth).

### Differential gene expression analysis

The bam files gained from the mapping procedure were further processed by the statistical program R. In a first step, the reads mapping as unique against a gene were counted. The annotation for *C. glutamicum* genome were extracted from the reference genome. The raw-count table was further processed with the DESeq function of the DeSeq2 package (version 1.18.1) to obtain gene expression data. Only genes with an adjusted *p*-value > 0.05 were considered to be differentially expressed.

### Read coverage analysis

For each base position, coverage statistics were obtained from the samtools depth function. The program R was used to handle large data sets and to calculate the average base coverage and reduce the data points further for the coverage of every gene. The program GraphPad Prism 7 was used to visualize the data sets.

## Electronic supplementary material

Supplementary Information

Table S2

Table S3

## Data Availability

All data generated or analyzed during this study are included in this published article (and its Supplementary Information files) or will be provided by the corresponding authors upon request. RNA-seq data have been deposited in the ArrayExpress database at EMBL-EBI (www.ebi.ac.uk/arrayexpress) under the accession number E-MTAB-6809 (https://www.ebi.ac.uk/fg/annotare/edit/6434/).
