# Supplementary material for: Cytometry meets next-generation sequencing – RNA-Seq of sorted subpopulations reveals regional replication and iron-triggered prophage induction in Corynebacterium glutamicum
Source: Sci Rep. 2018 Oct 5;8:14856. doi: 10.1038/s41598-018-32997-9 (PMC6173762; doi:10.1038/s41598-018-32997-9)
Supplement: Supplementary file 1 — Supplementary Information [file 41598_2018_32997_MOESM1_ESM.pdf]

Supplementary Information to:

Cytometry meets next-generation sequencing – RNA-Seq of sorted subpopulations reveals regional replication and iron-triggered prophage induction in *Corynebacterium glutamicum*

Raphael Freiherr von Boeselager, Eugen Pfeifer, Julia Frunzke*

Table S1: Oligonucleotides used in this study.

**pJC1_P$_{divS}$-*venus***	fw_PdivS(cg2113)	GCG ACG CCG CAG GGG GAT CCG CTA GCC TCG TGC ACC TGC TGG CTT C	PCR product contains overlapping sequences to the pJC1-venus-term plasmid *BamHI* restricted and the PCR product below; template genomic *C. glutamicum* DNA
	rv_PdivS(cg2113)	CAC CAT GAT ATC CCT CCT CTA ATC TAG CTC TTT ACC CGC ATA AAC	
	rv_pJC1_OL_venus	AAA ACG ACG GCC AGT ACT AGT TAC TTG TAC AGC TCG TCC ATG C	PCR product contains overlapping sequences to the pJC1-venus-term plasmid *SpeI* restricted and the PCR product above; template plasmid carrying *venus*
	fw_pJC1_OL_venus	TAG AGG AGG GAT ATC ATG GTG AGC AAG GGC GAG GAG C	
**pAN6-*dtxR***	dtxR_fw	TGCAGAAGGAGATATACATAGTGAAGGATCTGGTCGATACCAC	PCR product (*dtxR* gene, 687 bp) contains overlapping sequences (20 bp at each end) to *Eco*RI, *Nde*I digested pAN6
	dtxR_rv	AAAACGACGGCCAGTGAATTTTAGCCCTCAACCTTTTCTACGCG	
**pAN6-*recA***	recA_fw	TGCAGAAGGAGATATACATAATGGCTCCCAAGAAGACAGCAAC	PCR product (*recA* gene, 1131 bp) contains overlapping sequences (20 bp at each end) to *Eco*RI, *Nde*I digested pAN6
	recA_rv	AAAACGACGGCCAGTGAATTTTAGTCTTCAGCGTCTGCTTCGG	

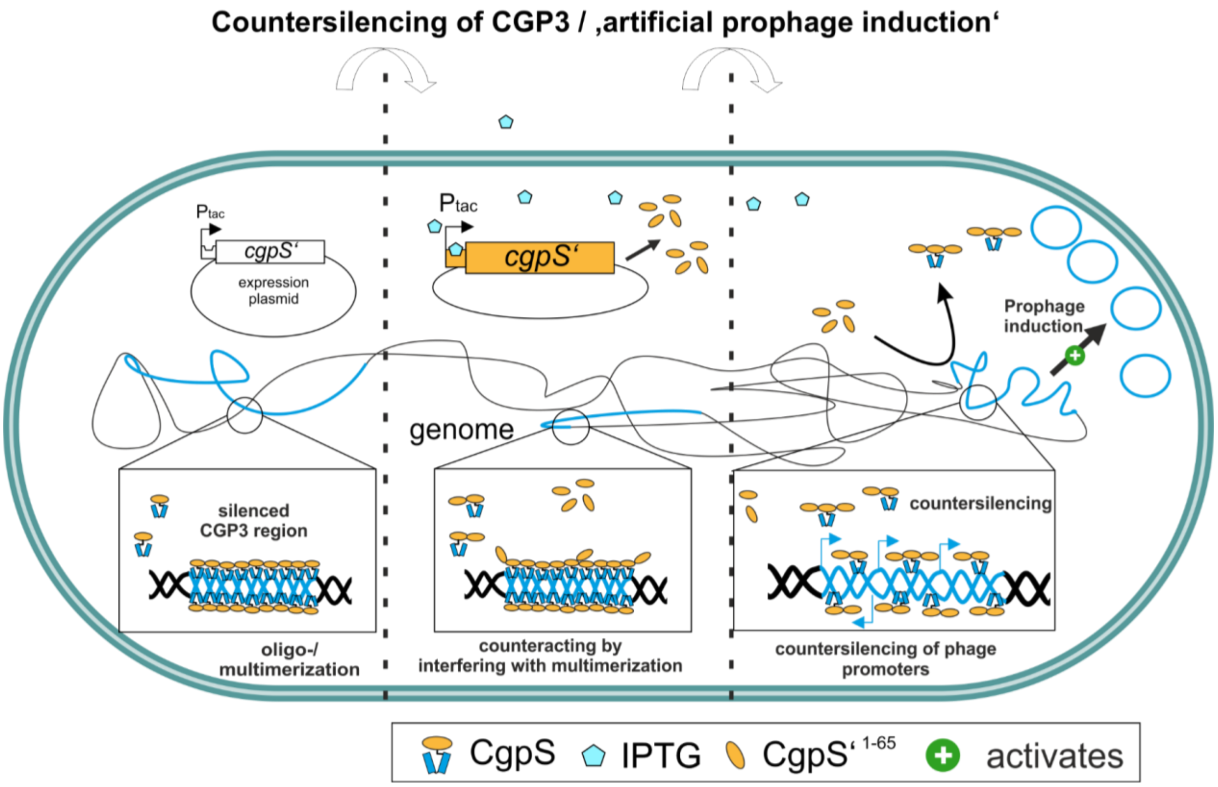

Figure S1: Countersilencing of CGP3 induction by overproduction of the N-terminal oligomerisation domain of CgpS. Recently, we described the xenogeneic silencer CgpS which acts as a silencer of phage gene expression in *C. glutamicum* (left panel, Pfeifer et al., 2016). As shown in the previous study, production of a truncated CgpS variant, lacking its DNA binding domain, will counter act CgpS-mediated silencing and finally lead to prophage induction (right panel). In this study, we used this approach to modulate the fraction of prophage induced *C. glutamicum* cells for the establishment of the RNA-Seq workflow.

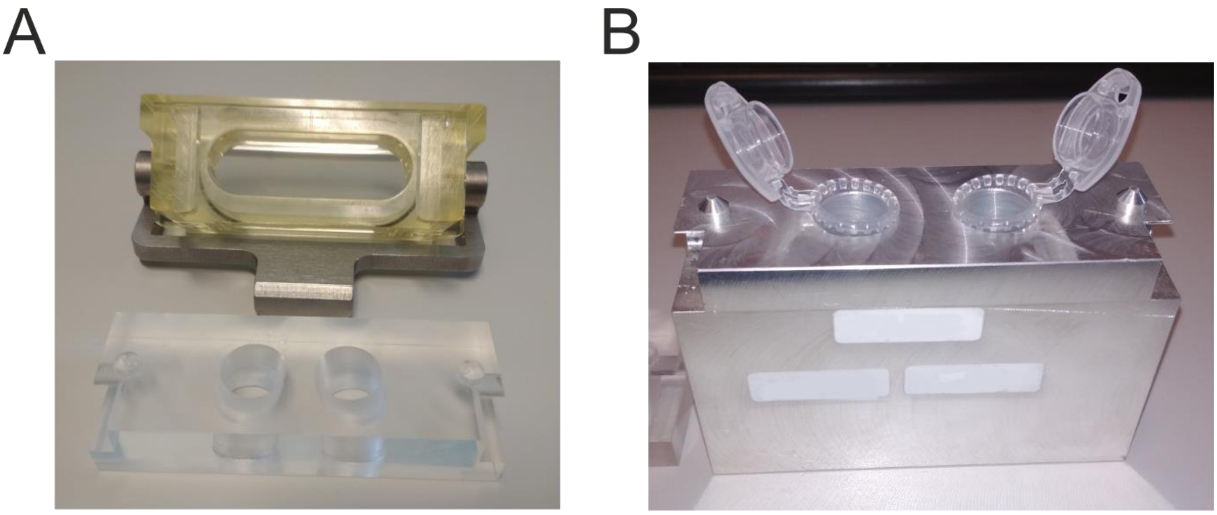

Figure S2: Custom-made sorting racks to optimize cell sorting via FACS. Both sorting rack systems fit in the original tube holder system of a FACS ARIA II (BD); two 5 ml tubes can be inserted in the rack. **A** The original tube holder is shown above and below the actual version of the sorting rack used for sorting cells in stabilisation agents. This shortened rack version allows to place a magnetic stirrer below the tubes to immediately disperse sorted cells in the stabilisation agent. **B** The cooling rack was stored at -80°C prior sorting. Cells were sorted into 5 ml tubes and directly frozen during the sorting procedure.

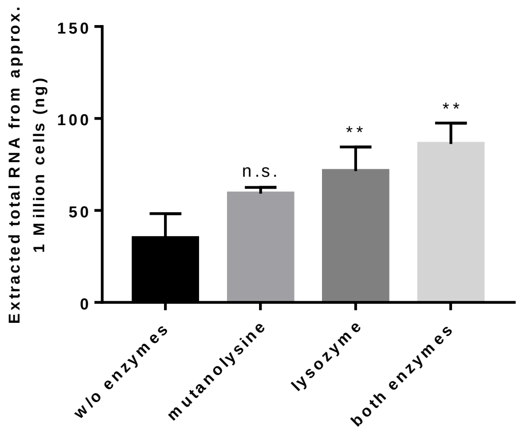

Figure S3: Enzymatic treatment increases the extraction of total RNA. Approximately one million cells were incubated either with chicken egg lysozyme or mutanolysine from *Streptomyces globisporus*. After 15 minutes the RNA was extracted with NucleoZol and measured with a NanoDrop. The bar plot represents average data including standard deviation of three biological replicates (students t-test analysis against the control data set w/o enzymes; *p*-value ≥ 0.05 were considered as not significant (n.s.); ** *p* ≤ 0.01).

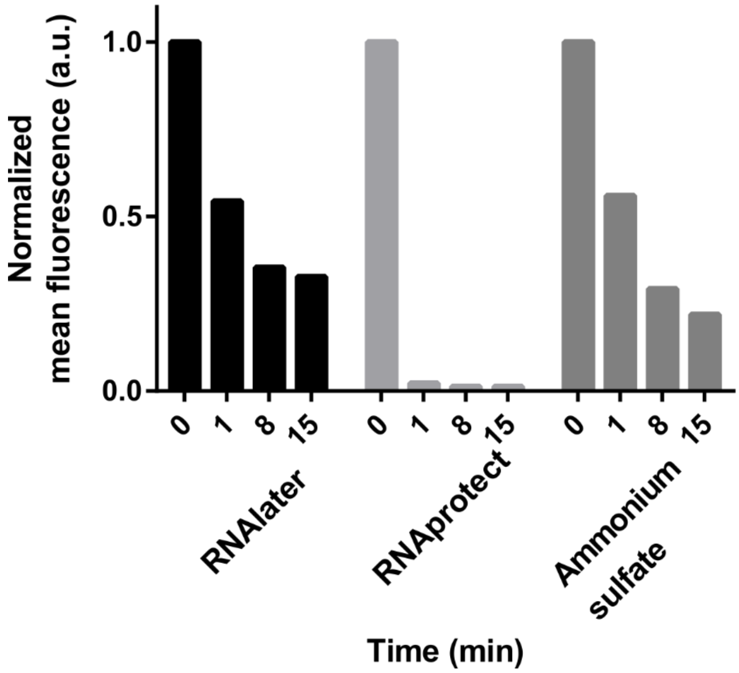

Figure S4: Quenching effect of stabilisation agents to fluorescent output of *C. glutamicum* cells. *C. glutamicum::Plys-eyfp* / pAN6_*N-cgpS* cells were cultivated in CGXII medium with 150 µM IPTG. After 6 hours, the sample was diluted (1:3) in one of the stabilisation agents and the fluorescence was measured in a flow cytometer. When the cells were stored in RNAlater, the fluorescence intensity drops to half and is further decreased to approximately 30% in 15 minutes. An ammonium sulfate solution (25 mM sodium citrate, 10 mM EDTA, 70 g ammonium sulfate/100 ml, pH 5.2) with a similar composition as RNAlater showed a similar effect. However, RNAprotect abolished the fluorescence directly after adding the solution to the cells. In all cases, the stabilization agent had a significant effect on the fluorescent output of the cells. Therefore, addition of the stabilization agent prior to cells sorting appears not to be recommendable.

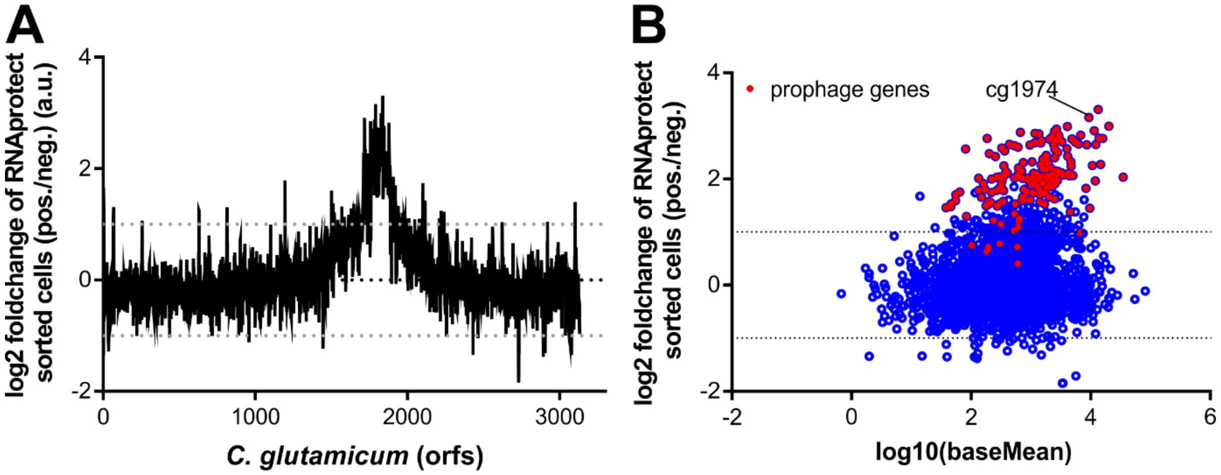

Figure S5: Differential gene expression analysis of cells sorted into RNAprotect. *C.glutamicum*::*Plys-eyfp* / pAN6_*N-cgpS* cells were cultivated in CGXII medium with 2 % glucose and 150 µM IPTG. After six hours the cells were sorted using FACS (gating strategy in Figure 3), further processed and sequenced (Figure 2 and material and methods). **A/B** The log2(foldchange) was calculated for each gene. The major peak represents the CGP3 region. Also the neighbouring regions showed a slightly increased coverage (see Figure 4 for details on regional replication).

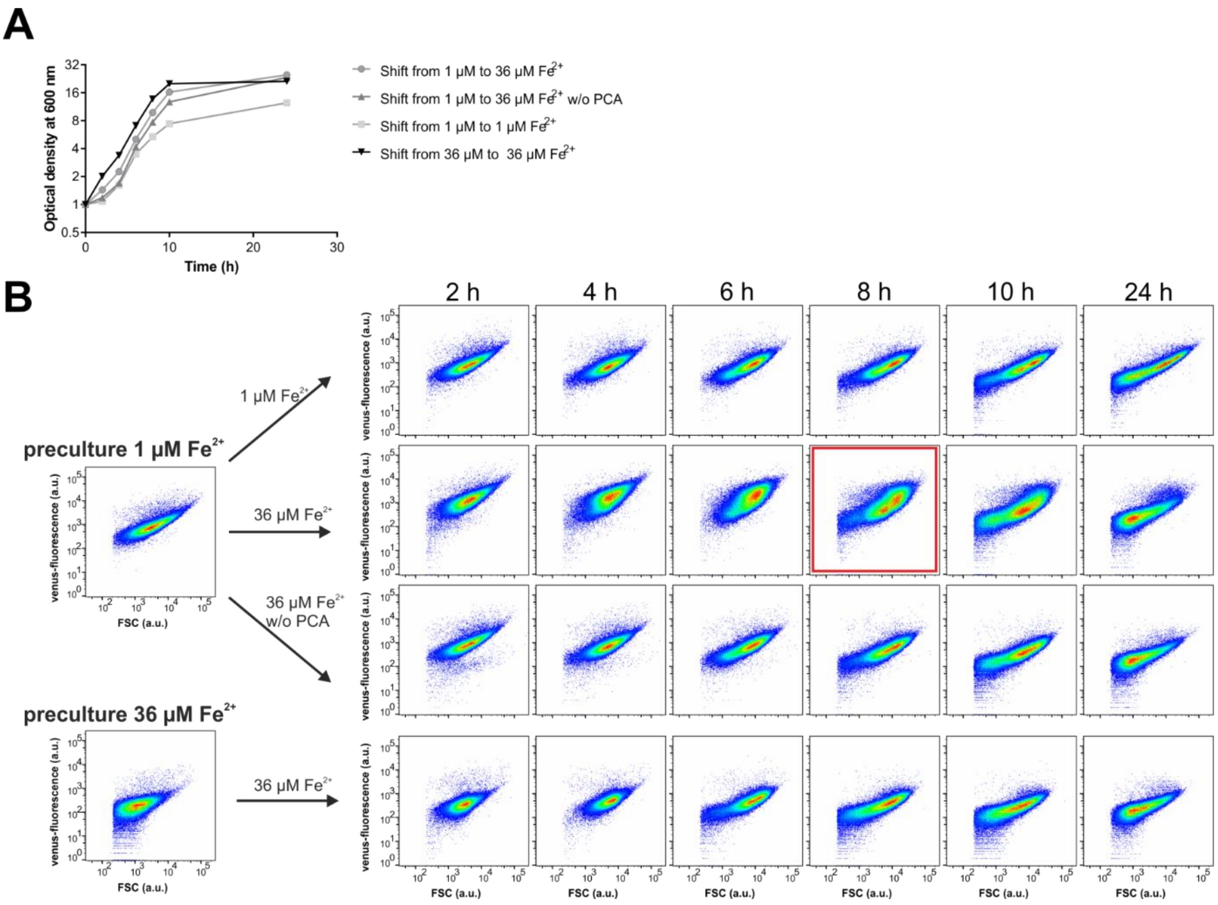

Figure S6: An upshift in iron concentration causes induction of the SOS response (P_{divS}) in the early exponential growth phase of *C. glutamicum*. In this experiment, *C. glutamicum*/ pJC1_*PdivS-venus* cells were cultivated in CGXII medium with 2 % (w/v) glucose under low iron (1 µM $FeSO_4$) and standard (36 µM $FeSO_4$) conditions. Cells pre-cultivated under low iron were transferred into fresh CGXII medium with either 1, 36 µM $FeSO_4$ or 36 µM $FeSO_4$ without protocatechuate (added as iron chelator in standard CGXII medium). As control, cells cultivated in 36 µM $FeSO_4$ were transferred in fresh medium with 36 µM iron. Samples were taken and analysed by flow cytometry to monitor the output of the P_{divS} reporter. When cells were shifted from 1 µM to 36 µM $FeSO_4$, a subpopulation appeared which showed an increased P_{divS} reporter output after 6 hours of cultivation. When shifting from 1 µM to 36 µM iron without PCA or from 36 µM to 36 µM $FeSO_4$, no significant increase of the reporter output was observed.
